# The Zinc-Finger Protein SOP1 Is Required for a Subset of the Nuclear Exosome Functions in Arabidopsis

**DOI:** 10.1371/journal.pgen.1005817

**Published:** 2016-02-01

**Authors:** Kian Hématy, Yannick Bellec, Ram Podicheti, Nathalie Bouteiller, Pauline Anne, Céline Morineau, Richard P. Haslam, Frederic Beaudoin, Johnathan A. Napier, Keithanne Mockaitis, Dominique Gagliardi, Hervé Vaucheret, Heike Lange, Jean-Denis Faure

**Affiliations:** 1 Institut Jean-Pierre Bourgin, INRA, AgroParisTech, CNRS, Université Paris-Saclay, Versailles, France; 2 Center for Genomics and Bioinformatics, Indiana University, Bloomington, Indiana, United States of America; 3 School of Informatics and Computing, Indiana University, Bloomington, Indiana, United States of America; 4 Univ Paris-Sud, Université Paris-Saclay, Orsay, France; 5 Department of Biological Chemistry and Crop Protection, Rothamsted Research, Harpenden, Herts, United Kingdom; 6 Department of Biology, Indiana University, Bloomington, Indiana, United States of America; 7 Pervasive Technology Institute, Indiana University, Bloomington, Indiana, United States of America; 8 Institut de Biologie Moléculaire des Plantes, Centre National de la Recherche Scientifique, UPR 2357, Université de Strasbourg, Strasbourg, France; University of California Riverside, UNITED STATES

## Abstract

Correct gene expression requires tight RNA quality control both at transcriptional and post-transcriptional levels. Using a splicing-defective allele of *PASTICCINO2* (*PAS2*), a gene essential for plant development, we isolated suppressor mutations modifying *pas2-1* mRNA profiles and restoring wild-type growth. Three suppressor of pas2 (*sop*) mutations modified the degradation of mis-spliced *pas2-1* mRNA species, allowing the synthesis of a functional protein. Cloning of the suppressor mutations identified the core subunit of the exosome SOP2/RRP4, the exosome nucleoplasmic cofactor SOP3/HEN2 and a novel zinc-finger protein SOP1 that colocalizes with HEN2 in nucleoplasmic foci. The three SOP proteins counteract post-transcriptional (trans)gene silencing (PTGS), which suggests that they all act in RNA quality control. In addition, *sop1* mutants accumulate some, but not all of the misprocessed mRNAs and other types of RNAs that are observed in exosome mutants. Taken together, our data show that SOP1 is a new component of nuclear RNA surveillance that is required for the degradation of a specific subset of nuclear exosome targets.

## Author Summary

Cells use various RNA quality control mechanisms to monitore the correct expression of their genome. Indeed, gene transcription can often generate faulty transcripts that are rapidly degraded to avoid possible deleterious effects to the cell. RNA degradation by the exosome is the main pathway for the removal of unwanted RNA in all kingdoms. Recognition of aberrant RNA involves a number of RNA binding proteins and other factors that target them for degradation by the exosome. Here, we used a genetic approach to identify proteins involved in the degradation of a mis-spliced RNA by the nuclear exosome in plants. Our screen identified two known components of nuclear RNA degradation pathway, namely the exosome core subunit RRP4 and the exosome-associated RNA helicase HEN2 that is required for the elimination of non-ribosomal RNAs by the nuclear exosome. Furthermore, we identified SOP1 as a novel putative exosome cofactor that is required for the degradation of some, but not all, of the substrates of HEN2.

## Introduction

The synthesis of mRNA in eukaryotes is a complex multistep process, involving the transcription of DNA into RNA, capping, splicing of intronic sequences and maturation of the 3’ end of the messenger prior to export to the cytoplasm for translation into protein. Production of functional RNA can be impaired by either genetic mutation or incorrect processing; both can be deleterious for the cell and have been associated with various human diseases [1,2]. To prevent the production of potentially harmful RNA, eukaryotic cells employ numerous RNA surveillance mechanisms enabling the recognition and degradation of defective or aberrant RNA and thereby ensure quality control throughout the RNA production pipeline [3–5].

One of the principal contributors to RNA surveillance and quality control is the RNA exosome, a multi-subunit complex that provides the main 3’-5’ exoribonuclease activity in eukaryotic cells [6–8]. The exosome complex consists of a core complex of nine conserved proteins and associated ribonucleases. In addition, the exosome interacts with activator/adaptor complexes containing RNA helicases, RNA binding proteins or terminal nucleotidyl transferases that are required for exosome activity and are involved in substrate recognition. The composition of these activator/adaptor complexes varies between different intracellular compartments and also between species. In mammals, the nucleolar exosome complex interacts with the RNA helicase MTR4, the RNA binding protein ZCCHC7, and the terminal nucleotidyl transferase hTRF4 in a complex similar to yeast TRAMP complexes [9]. The human MTR4 is also present in the nucleoplasm where it is associated with the RNA binding proteins ZCCHC8 and RBM7 to form the so-called NEXT (Nuclear EXosome Targeting complex) complex [10,11]. NEXT targets promoter upstream transcripts, enhancer RNAs, 3’ extended small nucleolar RNAs (snoRNAs) and introns and is considered as a central activator/adaptor complex of exosome-mediated RNA surveillance.

The core exosome and many of its cofactors are conserved in plants [12–14]. In Arabidopsis (*Arabidopsis thaliana*), the nucleolar exosome is bound to AtMTR4, which in turn associates with ribosome biogenesis factors [14,15]. The nucleoplasmic exosome associates with HUA-ENHANCER2 (HEN2), an RNA helicase closely related to MTR4. HEN2 is part of a NEXT-like complex and required for the elimination of virtually all types of non-ribosomal exosome substrates including snoRNAs, a range of other non-coding RNAs and 3’ or 5’ extended mRNAs [14]. Downregulation of HEN2 also results in the accumulation of transcripts comprising exons and unspliced introns, suggesting that HEN2 targets also alternatively or mis-spliced mRNAs for degradation by the exosome. Hence, HEN2 appears to be the general cofactor of nuclear RNA surveillance in Arabidopsis.

Here, we report the identification of SOP1, a zinc-finger protein involved in nuclear RNA degradation. The *sop1* mutation suppresses the developmental phenotype of a splice site mutation in the essential *PAS2* gene. This splice site mutation results in the production of *pas2-1* mRNA variants that undergo degradation by the nuclear exosome. In *sop1 pas2-1* plants, selected *pas2-1* mRNA variants are stabilised, thereby allowing the production of a functional PAS2 protein. In addition, loss of *SOP1* results in the accumulation of splice variants generated from other gene loci, which also accumulate in *hen2* and exosome mutants. Similarly to exosome mutants, loss of SOP1 counteracts the posttranscriptional silencing of a transgene (PTGS), indicating that SOP1 contributes to RNA surveillance. However, only a portion of HEN2 targets accumulate in *sop1* mutants suggesting that SOP1 is involved in the degradation of only a subset of nuclear exosome targets.

## Results

### Isolation of *suppressor of pas2 (sop)* mutations restoring *pas2-1* developmental defects

**Fig 1:**
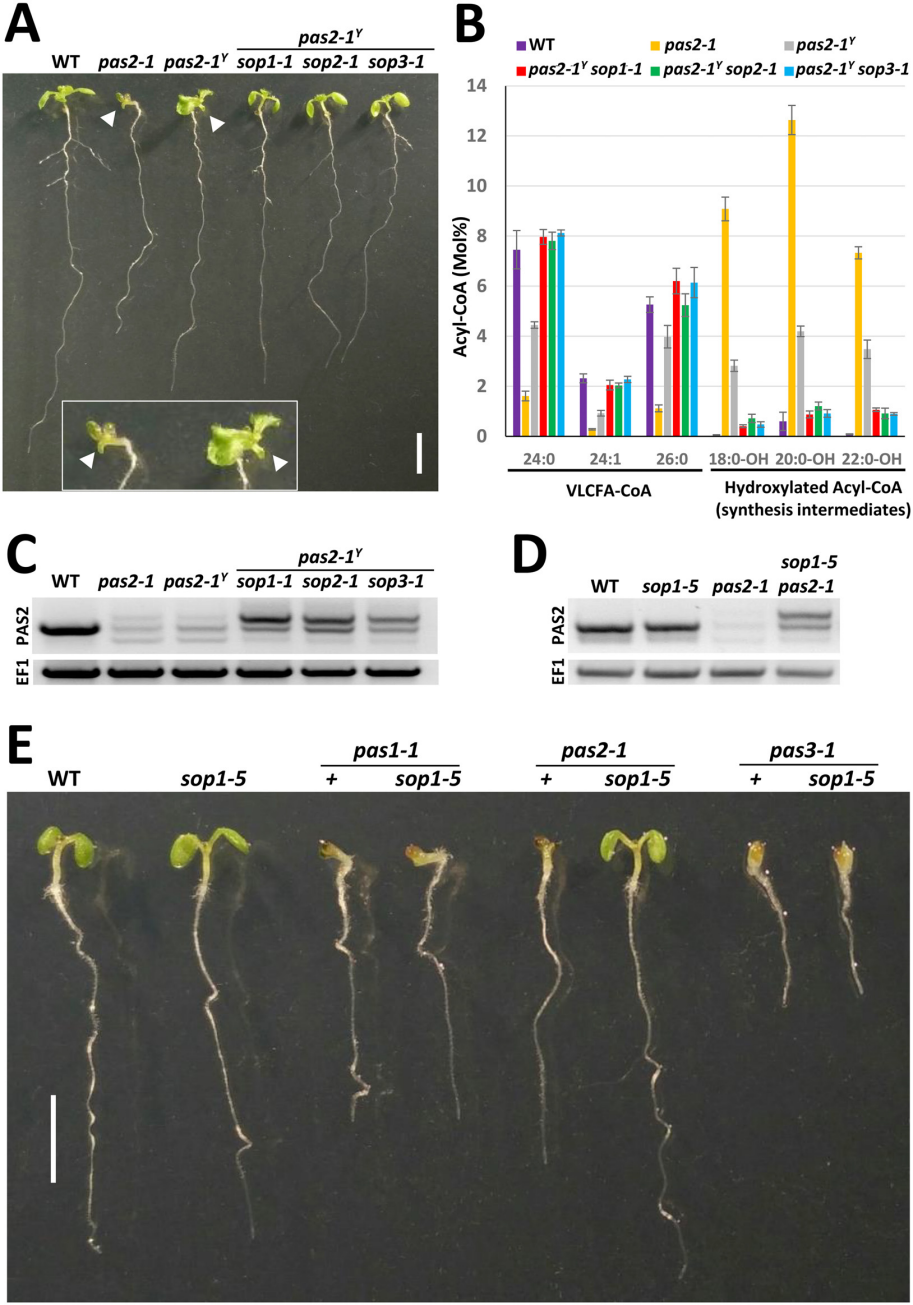
*sop* mutations suppress the *pas2-1* growth defect via restoration of Acyl-CoA dehydratase activity. (A) Picture of 12-day-old seedlings of the indicated genotype grown in petri dishes in long day conditions. Only *pas2-1* and *pas2-1*^*Y*^ presented misformed cotelydons (inset, white arrow head). Bar = 5mm. (B) Analysis of Acyl-CoA composition from the genotypes presented in 1A. Synthesis of very-long-chain-fatty-acids (VLCFA) is impaired in *pas2-1* mutants which accumulate hydroxylated synthesis intermediates. The phenotype is restored by the *sop* mutations. Error bars represent standard deviation (n = 3). (C) RT-PCR analysis of PAS2 mRNA splicing from the genotypes presented in 1A. (D) RT-PCR analysis of *pas2-1* mRNA isoforms accumulating in the indicated genotypes. The *sop1-5* mutant also accumulates the *PAS2-1*^*LONG*^ mRNA isoform. (E) Phenotype of 7-day-old seedlings of the indicated genotype showing that *sop1-5* specifically suppresses *pas2-1* but not any other VLCFA-deficient mutant.

*PAS2* (At5g10480) encodes the 3 hydroxy acyl-CoA dehydratase necessary for fatty acid elongation by the elongase complex in the endoplasmic reticulum [16]. The very long chain fatty acids (VLCFA; 20 carbons and over) produced by the elongase complex are essential for plant growth as demonstrated by the loss of PAS2 in *pas2* null mutants leading to embryo lethality [16]. However, the weak allele *pas2-1*, which harbors a point mutation affecting the splicing donor site of the eighth intron, allows viable embryogenesis and seedling development of the homozygous mutants [17,18]. The *pas2-1* homozygous mutant has a strong developmental phenotype with rod-shaped cotyledons and an enlarged hypocotyl due to an increased number of cell layers. The mutant plants also suffer from defective organogenesis with fused-organs, e.g. leaves, stems and flowers which leads to sterility [17]. During multiple rounds of mutant proliferation, we isolated a *pas2-1* homozygous natural variant that still showed the severe developmental *pas2* phenotype at the seedling stage, but developed into the adult stage and produced seeds. Importantly, this fertile variant, named *pas2-1*^*YaYa*^ (*pas2-1*^*Y*^) has the same genomic sequence of the *pas2-1* gene. The putative second site mutation or epigenetic phenomenon that underlies the partial restoration of the *pas2-1* phenotype in *pas2-1*^*Y*^ has not yet been identified. However, the restoration of fertility in *pas2-1*^*Y*^ made this natural variant an ideal starting point for a genetic screen to isolate supressors of the *pas2-1* seedling phenotype from an ethyl methane sulfonate (EMS) mutagenized population. Suppressor plants were screened from individual progeny of M1 plants at the seedling stage based on the restoration of cotyledon organogenesis of *pas2-1*^*Y*^ (Fig 1A and S1A Fig). We isolated eight *s**uppressors* *o**f* *p**as2* (*sop*) defining three complementation groups: four alleles for *sop1*, one allele for *sop2* and three alleles for *sop3* (S1A Fig). The three suppressors displayed almost wild type cotyledons and did not show any organ fusions, despite the presence of the splicing *pas2-1* mutation.

### *sop* mutations restore the defect of very-long-chain-fatty elongation caused by *pas2-1*

The loss of 3-hydroxy acyl-CoA-dehydratase activity in *pas2-1* mutants prevents the elongation of VLCFA with an acyl chain longer than 18 carbons [19,20]. In addition, loss of PAS2 activity results in the accumulation of 3-OH acyl-CoA intermediates [16] (Fig 1B and S1B Fig). To test if the suppression of *pas2-1* developmental defects in the isolated suppressor plants was caused by restoration of VLCFA content, we compared the acyl-CoA pools in wild type, *pas2-1*, *pas2-1*^*Y*^ and the suppressor plants. As compared to *pas2-1*, the *pas2-1*^*Y*^ plants showed a partial restoration of fatty acid elongation, but with the persistence of 3-OH acyl-CoA intermediates indicating that PAS2 dehydratase activity was still impaired in these plants (Fig 1B and S1B Fig). By contrast, all the *sop pas2-1*^*Y*^ suppressor lines had wild type levels of VLCFA, associated with an absence of detectable 3-OH acyl-CoA intermediates indicating a complete restoration of the acyl-CoA dehydratase activity (Fig 1B and S1B Fig). Since PAS2 provides the only acyl-CoA dehydratase activity in plants [16], these results indicated that the suppression of the *pas2-1* phenotype in *sop* lines was achieved by restoration of PAS2 activity.

### *sop1* specifically suppresses the splicing-defective *pas2-1* allele

Next, we tested whether the *sop1* mutation suppresses specifically the *pas2-1*^*Y*^ phenotype or can also suppress the phenotype of other VLCFA-deficient mutants. For this purpose, we introgressed *sop1-5*, a knock-out allele, that harbours a T-DNA insertion in the At5g21580 locus which encodes the SOP1 protein (see below), into the original *pas2-1* mutant as well as into *pas1-2*, *pas2-4* and *pas3-1* mutants [16,21,22]. Importantly *sop1-5* suppressed the *bona fide pas2-1* mutant (Fig 1E). Hence suppression of the *pas2-1* phenotype by *sop1* does not require the presence of the *pas2-1*^*yaya*^ background and is caused by the loss of SOP1/At1g21580 function. By contrast, *sop1-5* did not suppress VLCFA deficient *pas1* and *pas3* mutants (Fig 1E), indicating that *sop1* is not a general suppressor of VLCFA deficiency. Moreover, *sop1-5* was also unable to suppress the embryo lethality of a *pas2-4* knock-out mutant, as no homozygous *pas2-4* could be recovered from 24 F3 plants from the progeny of a *pas2-4* +/- *sop1-5* -/- parental plant (Fisher’s exact test, p = 0.0219). Thus *sop1* specifically suppresses the *pas2-1* mis-spliced allele, but does not compensate for a complete loss of *PAS2* function.

### *sop* suppressors accumulate different levels of *pas2-1* splice isoforms

Knowing that the *pas2-1* allele harbors a point mutation affecting the splicing donor site of the eighth intron, we reasoned that the suppression of *pas2-1* by *sop* mutations could be due to a restoration of the splicing defect. To test this hypothesis, we analyzed *pas2-1* mRNA produced in the suppressor background by RT-PCR (Fig 1C and 1D). While a single band was obtained from WT plants, three bands were detected in *pas2-1*, *pas2-1*^*y*^ and all three *sop pas2-1* double mutants. This result indicates that the splicing defect of the *pas2-1* mutant was not restored in *pas2-1*^*y*^ or in the *sop* mutants. On the contrary, an accumulation of the largest splicing isoform was observed. When compared to *pas2-1*, *pas2-1*^*Y*^*sop* suppressor plants had also slightly higher levels of the *PAS2-1* mRNA of wild type size, albeit at much lower levels than WT plants (Fig 1C and 1D). An identical repartition of *PAS2* mRNA isoforms was observed when *sop1-5* was introgressed in the *pas2-1* background, while the *sop1-5* mutation alone did not alter the expression of *PAS2* mRNA in WT background (Fig 1D and S2B Fig). These data suggested that the *sop* mutations affect the production or the stability of specific mRNA isoforms generated from the *pas2-1* locus.

To understand the splicing defects present in *pas2-1* mutants, we cloned and sequenced the *PAS2-1* RT-PCR products. Four different isoforms were identified (for sequence detail see S3 Fig). The longest isoform (*PAS2-1*^*LONG*^) corresponded to an incompletely spliced *PAS2* mRNA, which retained the 8th intron leading to the production of an mRNA with a premature termination codon (PTC). The shortest (*PAS2-1*^*SHORT*^) PCR product lacked the 8th exon resulting in a direct fusion of Exon 7 to Exon 9, which results in a frame shift leading to the loss of the stop codon. The band with a size similar to wild type corresponded to a mix of two isoforms. One corresponded to a mispliced isoform (*PAS2-1*^*MIDb*^) that used a cryptic splicing donor site (GT) seven nucleotides upstream of the *pas2-1* mutation, and also resulted in the loss of a stop codon. The second product present in the WT-size band corresponded to a correctly spliced *PAS2-1* mRNA (*PAS2-1*^*MIDa*^) which retained the point mutation present in the *pas2-1* allele resulting in a single amino-acid change in the PAS2 protein sequence (Gly^199^Ser). This latter isoform is the only isoform that is predicted to produce a full-length protein.

### Higher levels of a specific *pas2-1* splice isoform account for the restoration of PAS2 activity in *sop1 pas2-1* plants

**Fig 2:**
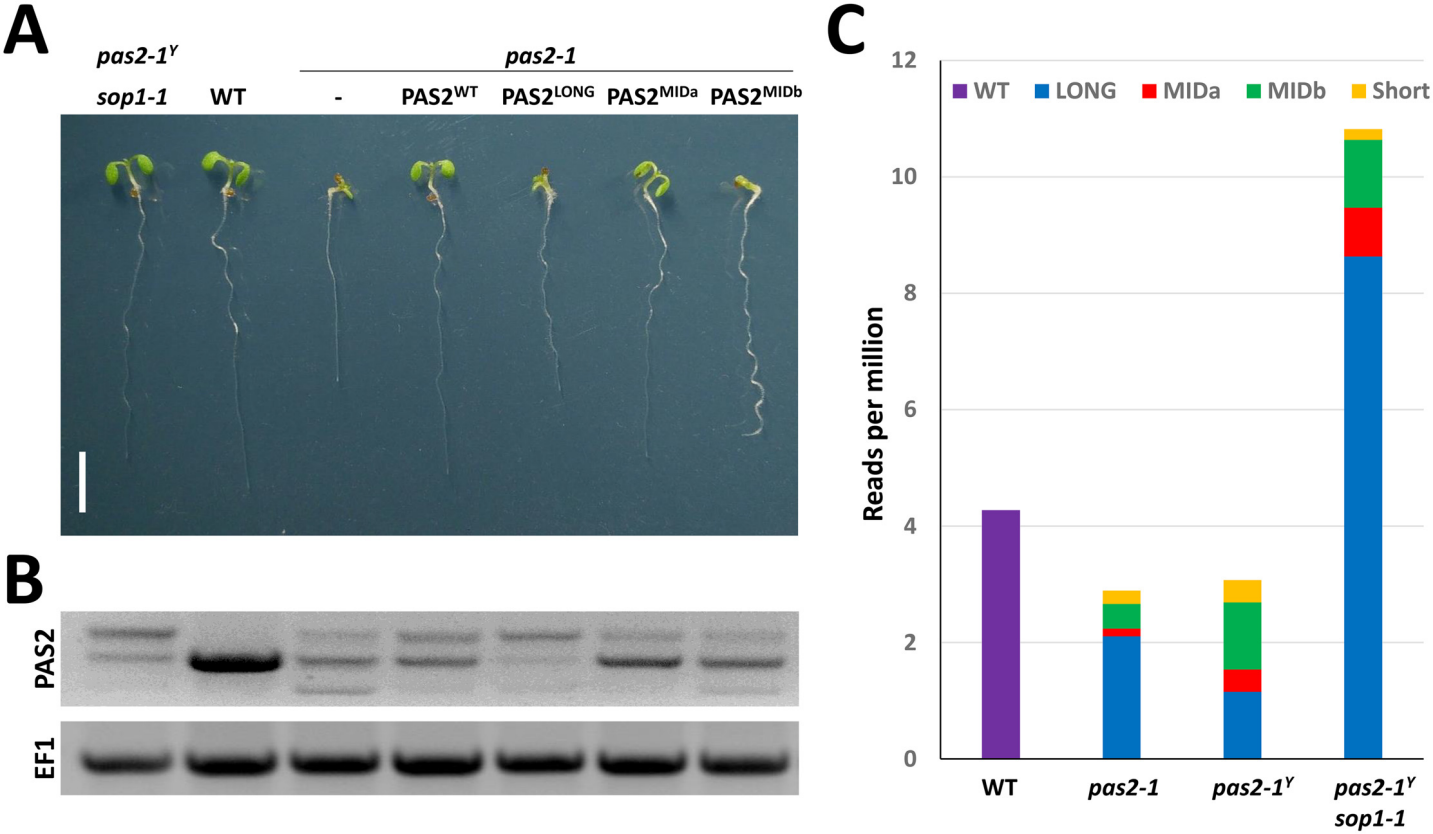
*sop1* accumulates different levels of *pas2-1* splice isoforms, some of which encode a functional PAS2 protein. (A) Phenotype of 7-day-old *pas2-1* seedlings expressing wild-type *PAS2* or the various *PAS2-1* isoforms under the control of the endogenous PAS2 promoter. Only the expression of *PAS2*^*WT*^ and *PAS2-1*^*MIDa*^ isoforms complement the growth defect of *pas2-1*. (B) RT-PCR analysis of *PAS2* mRNA isoforms in the seedlings presented in 2A. (C) Quantification of RNA-seq reads matching the different *pas2-1* mRNA isoforms produced in 12-day-old seedlings of the indicated genotypes. The values are normalized by the number of reads per million reads. Details regarding the *pas2-1* mRNA isoforms and the corresponding specific consensus sequences can be found in S3 Fig.

To investigate whether the enhanced level of one of the *pas2-1* splicing variants could confer suppression, we expressed the different RNA isoforms under the endogenous PAS2 promoter in a *pas2-1* mutant background. Beside wild type PAS2 protein, only its closest isoform PAS2-1^MIDa^ was able to complement *pas2-1* mutant (Fig 2A), suggesting that PAS2^G199S^ encoded by *PAS2-1*^*MIDa*^ RNA is a functional dehydratase.

The relative levels of the different mRNA isoforms present in WT, *pas2-1*, *pas2-1*^*y*^ and *pas2-1*^*y*^*sop1-1* plants were estimated with the number of RNAseq reads matching a ten nucleotide long sequence spanning the exon junction involved in each of the *pas2-1* mRNA isoforms (S3C Fig, sequences in bold). In agreement with the RT-PCR results (Fig 1D), the quantification of RNA seq reads showed that the *PAS2-1*^*LONG*^ isoform was the most abundant isoform in *pas2-1*^*Y*^*sop1-1* (Fig 2C, 7.5-fold increase compared to *pas2-1*^*Y*^). Interestingly, the higher levels of the *PAS2-1*^*LONG*^ RNA were associated with a mild increase of the *PAS2*^MIDa^ (2.17-fold), but not *PAS2*^*MIDb*^ RNA (1.01-fold). While the ratio of *PAS2*^*MIDa*^/*PAS2*^*MIDb*^ was about 0.3 in *pas2-1* and *pas2-1*^*Y*^, it raised to 0.7 in *pas2-1*^*Y*^*sop1-1* thanks to the accumulation of *PAS2*^*MIDa*^. These data indicate that the restoration of acyl-CoA dehydratase activity in *sop1* plants was due to higher levels of the *PAS2-1*^*MIDa*^ compared to *pas2-1* and *pas2-1*^*Y*^ plants, which in turn led to the production of a functional PAS2^G199S^ protein. Furthermore, our data suggest that that *sop1* favours the production of the *PAS2*^MIDa^ either directly by affecting the efficiency of *pas2-1* splicing, or indirectly by stabilising the intron-retaining RNA isoform *PAS2-1*^*LONG*^, which in turn would improve the production of *PAS2-1*^*MIDa*^ isoform.

### *sop1* is not a general suppressor of splice site mutations

**Fig 3:**
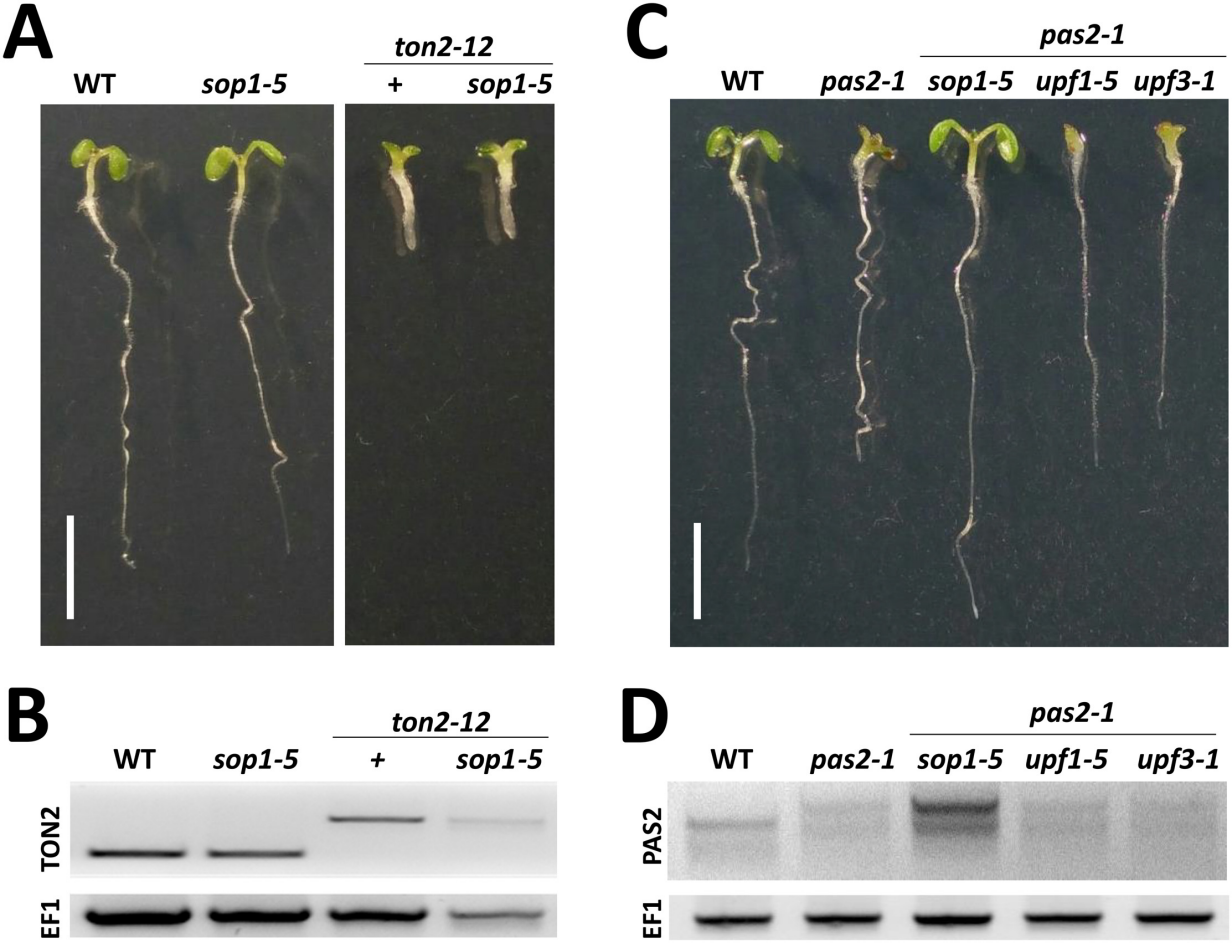
*sop1* do not suppress other splice mutants and is independent of the NMD pathway. (A) Phenotype of 7-day-old seedlings of *ton2-12* alone or in combination with *sop1-5*. SOP1 loss of function does not suppress the phenotype caused by the mis-spliced *ton2-12* allele. (B) RT-PCR analysis of *ton2-12* mRNA isoforms in the indicated genotypes. The *sop1-5* mutant did not induce the accumulation of mispliced *ton2-12* mRNA. (C) Phenotype of 7-day-old seedlings of the indicated genotype showing specific suppression of *pas2-1* by *sop1-5* but not by non-sense mediated decay *upf1-5* and *upf3-1* mutants. (D) RT-PCR analysis of *PAS2* mRNA isoforms in the indicated genotypes showing that the *PAS2-1*^*LONG*^ isoform is not targeted for NMD.

To ascertain whether *sop1* can affect the levels of mRNA isoforms generated from other splicing-defective loci, we crossed *sop1-5* to *ton2-12*, a mutant harbouring a mutation in a splicing donor site (GT->AT of the first intron) of the *TONNEAU2* (*TON2*) gene, encoding the regulatory subunit of the protein phosphatase 2A (PP2A) complex involved in the control of the orientation of the division plane [23]. The *ton2-12* mutation results in the production of an mRNA isoform with similar features to *pas2-1* (retained intron with PTC) and also leads to a strong developmental phenotype [23]. However, *sop1-5* did not rescue the growth defect of *ton2-12* mutants (Fig 3A), and did not affect accumulation of the *ton2-12* intron-retaining RNA isoform (Fig 3B). We also queried intron-retention events in the *sop1-1* mutant in our RNAseq data to identify other mis-spliced RNA. In addition to the expected accumulation of introns corresponding to alternative splicing events, we identified only one locus (At5g36880) accumulating an intron specifically in *sop1-1* background. However, this intron retention was also associated with a point mutation of its 5’ intronic splice donor site in *sop1-1* (S4 Fig). Similarly to the *ton2-12* mutation, the intron-retaining transcript of At5g36880 did not accumulate in *sop1-1*. These results suggest that *sop1* influences *PAS2-1*^*LONG*^ mRNA accumulation, but does not have a general effect on the stabilisation of incompletely spliced mRNAs.

### Stabilization of intron-retaining *pas2-1* isoform in *sop* mutants does not require the NMD pathway

Our data indicate that the major effect of the *sop* mutations on *pas2-1* mRNA isoforms is the accumulation of the intron containing *PAS2-1*^*LONG*^ isoform (Figs 1D and 1E and 2C), suggesting that SOP1 affects either the production or the stability of this particular isoform. The *PAS2-1*^*LONG*^ isoform is characterised by two molecular determinants: the retained intron and the presence of a premature termination codon (PTC, S3C Fig), the latter of which is known to trigger rapid RNA degradation via the non-sense mediated mRNA decay (NMD) pathway [24]. Therefore, we tested the hypothesis that the *PAS2-1*^*LONG*^ isoform is a substrate for non-sense mediated mRNA decay [25–27]. The *pas2-1* mutant was crossed with mutants of *UPF1* (encoding an RNA Helicase) and *UPF3* (encoding an RNA-binding protein), both key components of the NMD pathway. The resulting double mutants were analysed for both growth and accumulation of the PAS2-1^LONG^ isoform. The results showed that neither *pas2-1 upf1-5* nor *pas2-1 upf3-1* double mutants suppressed the *pas2-1* growth phenotype (Fig 3C) or showed enhanced levels of the PTC containing *PAS2-1*^*LONG*^ RNA (Fig 3D). These results indicate that RNA degradation through NMD is not responsible for the low levels of *PAS2-1*^*LONG*^ isoforms observed in *pas2-1* mutants.

### The *SOP* loci encode proteins involved in RNA processing and degradation

**Fig 4:**
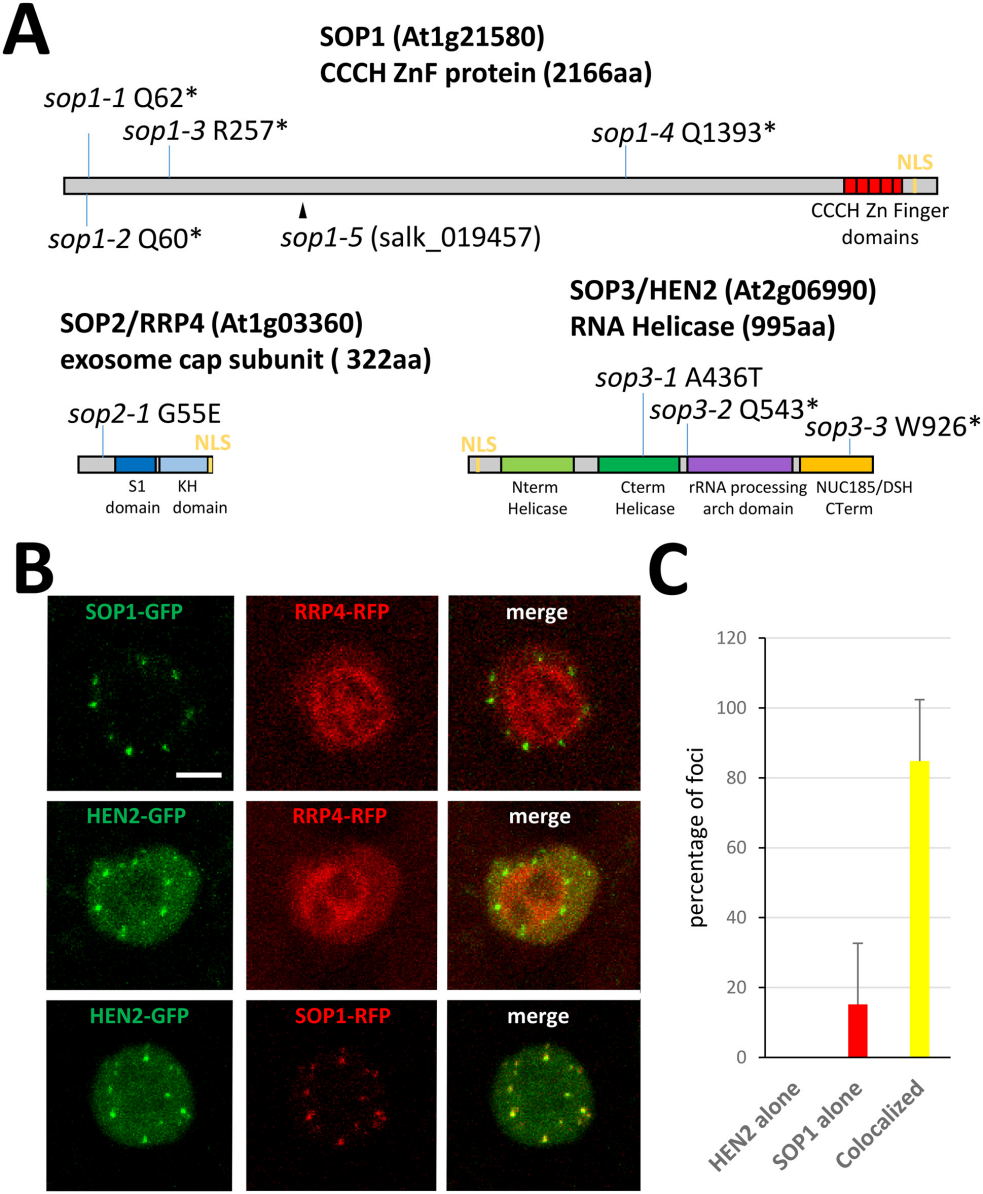
SOP proteins encode nuclear proteins involved in RNA quality control. (A) Diagram depicting protein identity and structure of SOP1, SOP2 and SOP3 with their color-coded annotated domains. Mutations characterized in this study are also displayed. (B) Confocal laser scanning imaging of root cells from plants stably co-expressing SOP1, SOP2 and SOP3 proteins in fusion with either GFP or RFP. (bar = 5μm). (C) Quantification of nuclear foci containing either HEN2 or SOP1 alone or both (n = 508 foci from 37 root epidermal cells).

To identify the *sop* mutations, we first conducted a positional cloning of the suppressor mutations with a mapping population prepared from a cross between the *pas2 sop* mutants (*Columbia* accession) and *Landsberg erecta* accession. In addition to the segregation bias on Chromosome V due to the presence of the *pas2-1* mutation (At5g10480), we identified 0.5-1Mb segregating regions on Chromosome I for *SOP1* or *SOP2* and Chromosome II for *SOP3*. Next generation sequencing of genomic DNA extracted from the suppressors *pas2-1*^*Y*^*sop1-1*, *pas2-1*^*Y*^*sop2-1* and *pas2-1*^*Y*^*sop3-1* identified the specific polymorphisms associated with each genotype and matching the coding sequence of genes present in the mapped regions of *SOP* loci (Fig 4A). For *sop1-1*, a unique single nucleotide polymorphism (SNP) in At1g21580 gene fulfilled these criteria and was further confirmed by sequencing three other alleles (all four *sop1* alleles contained PTC). Similarly, an SNP was found in At2g06990 gene for *sop3-1* and was confirmed with two other *sop3* alleles (one missense mutation and two PTC). For *sop2-1*, a candidate SNP in At1g03360 gene was identified and confirmed by complementation of the *pas2 sop2* mutant phenotype with the wild-type At1g03360 gene (S5B Fig).

Remarkably, all three SOP proteins are involved in RNA metabolism. SOP2 encodes Ribosomal RNA Processing 4 (RRP4), a core subunit of the RNA exosome required for the processing of rRNA, several snoRNA and the degradation of aberrant transcripts [12]. SOP3 encodes HUA-Enhancer 2 (HEN2), a RNA helicase homologous to MTR4, identified initially as a regulator of AGAMOUS splicing [28] and more recently as interacting with the nuclear exosome for the degradation of misprocessed mRNA and other types of non-ribosomal exosome targets [14]. SOP1 encodes a recently re-annotated large protein which was formerly annotated as two genes (At1g21570/AtC3H7 [29,30] and At1g21580, unknown protein). SOP1 contains five zinc-finger (ZnF) domains at its carboxy-terminus which may bind RNA [29].

### SOP1 and SOP3/HEN2 colocalize in nucleoplasmic speckles

While the exosome core complex is present in both nuclear and cytosol, HEN2 was shown to be a nuclear protein enriched in nucleoplasmic foci. We therefore compared the subcellular distribution of SOP proteins by expression of functional GFP fusion proteins in stable Arabidopsis transformants (S5A–S5C Fig). Confirming previous results, RRP4-GFP was detected in both the cytoplasm and nucleus, with a specific enrichment in the nucleoli (Fig 4B and 4C)[14,27], while HEN2-GFP was detected in nucloplasmic speckles, but also diffusely distributed in the nucleoplasm (Fig 4B and 4C)[14]. Interestingly, SOP1-GFP was not diffused in the nucleoplasm, but predominantly localized in nucleoplasmic speckles, similar to the foci labelled by HEN2-GFP (Fig 4B and 4C). Therefore co-localization of SOP1, SOP2/RRP4 and SOP3/HEN2 was assessed by co-expression of corresponding RFP and GFP fusion proteins. This experiment revealed that SOP1 indeed colocalized with SOP3/HEN2 in nucleoplasmic speckles while SOP2/RRP4 and SOP3/HEN2 colocalized diffusely in the nucleoplasm (Fig 4C). Those nucleoplasmic speckles were found throughout the nucleoplasm (S1 Movie) and presented a limited dynamic (S2 Movie) that was synchronous between SOP1 and HEN2 (S4E and S4F Fig). However, speckles containing exclusively SOP1 could also be occasionally observed (Fig 4C). These results reinforce the idea that SOP1 could be involved in similar functions than HEN2, namely the degradation of nuclear exosome targets.

### SOP1 partially overlaps with SOP2 an SOP3 in RNA processing and quality control

**Fig 5:**
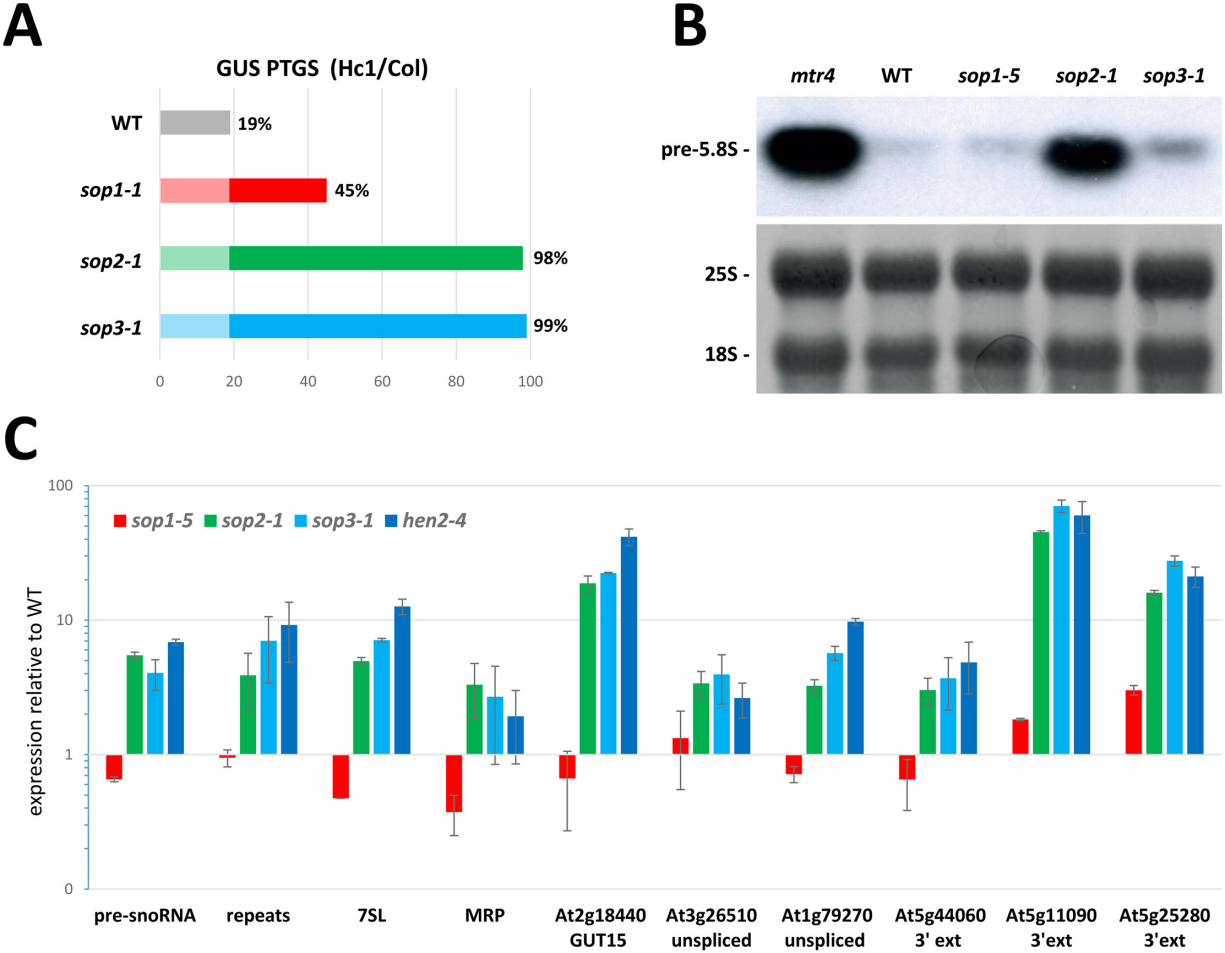
SOP1 partly overlaps with SOP2 and SOP3 in RNA processing and degradation. (A) Effect of *sop* mutations on post-transcriptional gene silencing (PTGS) of the 35S::GUS reporter line Hc1. Percentage represent the number of silenced plants (n = 96). (B) Northern blot showing the accumulation of 5.8S rRNA precurors in *sop1-5*, *sop2-1* or *sop3-1* single mutants compared to wild type (Col) and *mtr4*. The radiolabelled oligoprobe is complementary to the region directly 3’ of mature 5.8S rRNA. A methylene blue stain of the membrane is shown as loading control. (C) RT-qPCR analysis of transcript accumulation in *sop1-5*, *sop2-1* or *sop3-1* single mutants compared to wild type (Col 0) and *hen2-4*. Expression relative to wild type is presented on a log-scale, error bars represent SD (n = 2).

Defects in either nuclear or cytosolic RNA quality control (RQC) functions generally result in increased post-transcriptional (trans)gene silencing (PTGS). The rationale is that RQC serves as a first layer of defense to eliminate aberrant RNAs. Thus, aberrant transgene RNA bypass the RQC defenses and enter into the PTGS pathway only when the RQC machinery is dysfunctional or when it is saturated by a large excess of aberrant transgene RNA [14,27,31–34]. In particular, it was shown that mutations in the exosome core component RRP4 strongly enhance PTGS [27]. Mutations in HEN2, but not in MTR4, also strongly enhance PTGS, indicating that the degradation of abberant transgene RNA in the nucleus involves the nucleoplasmic fraction of the exosome [14]. The GUS tester line Hc1, which triggers PTGS in only 20% of the population at each generation [31,35], is a sensitive tool for monitoring the effect of both enhancers and suppressors of transgene PTGS. To quantify the effect of the *sop* mutations on PTGS, the Hc1 line was crossed to the three *sop* mutants and plants homozygous for both the transgene and the *sop* mutations were analyzed. As reported previously for *rrp4* and *hen2* mutants [14,27], PTGS was strongly enhanced in *sop2* and *sop3* mutants (Fig 5A). Interestingly, the *sop1* mutation also increased PTGS albeit to milder levels, suggesting that SOP1 is not essential, but indeed participates to RNA quality control.

To evaluate a possible role for SOP1 in RNA degradation by the nuclear exosome, we compared the accumulation of known exosome targets in *sop1*, *sop2 and sop3* mutants by Northern blots or qRT-PCR. In agreement with previous results [12,14], only *sop2/rrp4* mutants had elevated levels of 3’ extended pre-5.8S rRNA, a known target of the nucleolar exosome ([14,15] Fig 5B). By contrast, *sop1* did not accumulate 5.8S rRNA precurors similarly to *sop3/hen2* indicating that SOP1 is not involved in rRNA processing (Fig 5B). Among selected model targets of HEN2/SOP3 [14], *sop1* had an effect on one mis-spliced mRNA and two 3’ extended mRNAs (Fig 5C). However, the effect of *sop1* was weaker than the effect of *hen2/sop3-1*, a result corresponding to that observed for PTGS suppression (Fig 5A). Finally, unlike *hen2/sop3*, *sop1* mutants did not accumulate stable non-coding RNAs, precursors of snoRNAs, or transcripts generated from intergenic repeats (Fig 5C). Collectively these data suggested that SOP1 is dispensable for some of the reported functions of the nuclear exosome, but could be involved in the degradation of RNAs that are also substrates of the nucleoplasmic exosome and HEN2.

### SOP1 is required for the degradation of a subset of exosome targets

**Fig 6:**
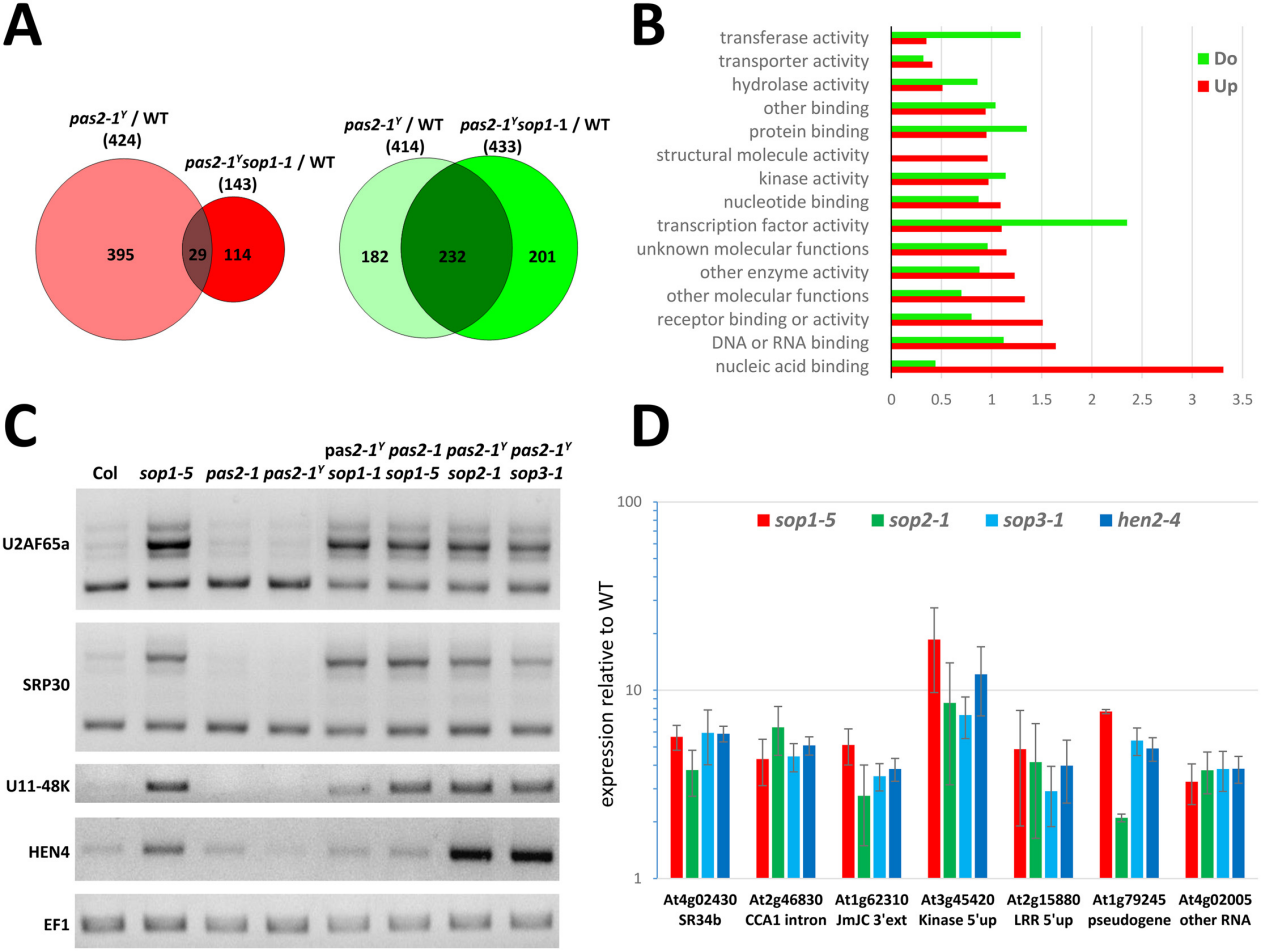
*sop1* accumulates a subset of exosome targets. (A) Venn Diagrams showing number of genes differentially expressed in the indicated genotypes analyzed by RNA seq. Upregulated genes (in red) or downregulated genes (in green) in *pas2-1*^*Y*^ and *pas2-1*^*Y*^*sop1-1* compared to wild type plants. (B) Functional classification based on the GO term « molecular process » of the 114 and 201 genes specifically up- or down-regulated in *sop1-1* as compared to wild type plants. (C) RT-PCR of selected *sop1*-dependent genes in the indicated genotypes. (D) RT-qPCR analysis of transcript accumulation in *sop1-5*, *sop2-1* or *sop3-1* single mutants compared to wild type (Col 0) and *hen2-4*. Expression relative to wild type is presented on a log-scale, error bars represent SD (n = 2).

To better understand the role of SOP1 in the accumulation of *pas2-1* mRNA and RNA quality control, we aimed to identify other transcripts affected by *sop1* mutation. Therefore, we compared the transcriptomes of WT, *pas2-1*^*Y*^ and *pas2-1*^*Y*^*sop1-1* plants by RNA seq. When comparing *pas2-1*^*Y*^ to wild type plants, 424 genes were induced more than 2-fold while 414 genes were repressed. Consistent with the full restoration of the VLCFA-deficiency in *pas2-1*^*Y*^*sop1* mutants (Fig 1B), the expression of most of these genes (93% and 44% for induced and repressed genes, respectively) was restored to wild type level in *pas2-1*^*Y*^*sop1* mutants. However, our analysis identified 114 and 201 genes that were specifically up- or down-regulated in presence of the *sop1* mutation (Fig 6A). Unlike *hen2* or exosome mutants, which were shown to accumulate a large number of non-genic transcripts [12,14], the majority of the transcripts that were misregulated in *sop1* were mRNAs (S1 Table) and likely include both direct targets of exosome-mediated degradation and secondary transcriptional responses. However, with the exception of the splicing factor SR34b (Fig 6B), which was reported to modulate the splicing of IRT1 (At4g19690, S1 Table, [36]), we did not identify obvious transcriptional cascades. Interestingly, a Go-term analysis revealed that many of the misregulated mRNAs in *sop1* are involved in splicing or other RNA-related processes (Fig 6B, S1 Table). Since some of the upregulated RNA processing or splicing factors identified by the RNA seq analysis were predicted to undergo alternative splicing, we evaluated the levels of splicing isoforms by RT-PCR (Fig 6C). For each of *HEN4* and *U11-48k* mRNAs, only one predominant splice form was detected but appeared to be more abundant in *sop1*, *sop2* and *sop3* mutants. For *SRP30* and *U2AF65a*, two main RNA isoforms were detected. While the levels of the smaller isoforms were similar in all samples, the larger isoforms generated by intron retention accumulated upon mutation of *SOP1*, *SOP2* and *SOP3* (Fig 6C). These data are in line with the idea that incompletely spliced mRNAs are targeted for exosome-mediated RNA degradation, and that *sop1* is involved in this process. As these alternatively spliced isoforms were not detected in NMD mutants (S6 Fig), their accumulation of in *sop1*, *sop2* and *sop3* is unlikely related to defects in non-sense mediated decay.

Finally, we analysed the upregulation of some of the candidate genes identified by RNA seq analysis by qRT-PCR in *sop1*, *sop2* and *sop3* mutants. For this experiment we used primer pairs located in the body of the mature RNA, but also primer pairs located in introns, or immediately upstream or downstream of the annotated mRNA, indicative of misprocessed mRNA with the typical features of *bona fide* exosome targets [14]. For all candidate targets tested, we detected a significant accumulation in *sop1*, *sop2* and *sop3* samples (Fig 6D). These data show that loss of *sop1* does indeed affect the degradation of a subset of exosome substrates, including misprocessed mRNA and transcripts expressed from pseudogenes and some non-coding loci.

To conclude, our data identify SOP1 as a Zn-finger protein that co-localises with the exosome-associated RNA helicase HEN2 and participates in the degradation of a selective subset of nuclear exosome targets including misprocessed mRNAs. Taken together, our results indicate that SOP1 functions as a co-factor of nuclear RNA quality control by the nucleoplasmic exosome.

## Discussion

In this study, we elucidated the molecular basis of the strong decrease in 3-hydroxy acyl-CoA dehydratase activity in the *pas2-1* mutant. In *pas2-1* plants, a mutation of the last nucleotide in the penultimate exon of *PAS2* (G^1841^A) prevent correct mRNA splicing leading to the retention of the last intron and to aberrant intron splicing donor site usage. This result in a low steady state levels of four different *pas2-1* mRNA isoforms, of which only *PAS2*^*MIDa*^ encodes a protein that retains 3-hydroxy acyl-CoA-dehydratase activity. Second site mutations in the exosome subunit RRP4 (in *sop2*), in the nuclear exosome cofactor HEN2 (in *sop3*) and in the Zn-finger protein SOP1/AT1G21580 (in *sop1*) result in the accumulation of the longest *PAS2-1* mRNA isoform, which still contains the unspliced 8th intron. In addition, *pas2-1 sop* double mutants have, relative to single *pas2-1* and *pas2-1*^*y*^ plants, higher levels of the functional PAS2-1^MIDa^ mRNA. These findings indicate that in *pas2-1*, the incompletely spliced *PAS2-1*^*LONG*^ isoform is recognized by the nuclear RNA surveillance machinery and targeted to rapid degradation by the nuclear exosome. Therefore, impaired RNA degradation in *pas2-1 sop* could lead to stabilisation of *PAS2-1*^*LONG*^ mRNA, allowing enough time for splicing to occur and resulting in an increased production of *PAS2-1*^*MIDa*^ mRNA to eventually produce an active PAS2-1 (Gly^199^Ser) protein. In other words, slowing down degradation could allow unefficient splicing to occur, as previously reported [37,38].

The *pas2-1* suppressor genetic screen identified two known components of the nucleoplasmic RNA surveillance machinery, HEN2/SOP3 and RRP4/SOP2, which confirmed the role of the exosome in the degradation of mispliced mRNAs. The G55E mutation in the *sop2* allele affects an evolutionary strictly conserved residue of the exosome core subunit RRP4 [39]. Based on the crystal structure of the yeast EXO9-RRP6 complex, this residue is located close to the N-Terminal Domain (NTD) of RRP4 which forms the interface of the core complex with RRP6 [40]. Interestingly, Arabidopsis has three RRP6 isoforms with different subcellular localizations [41]. However, none of these isoforms has yet been shown to interact with the core exosome [12,14]. Hence, we can only speculate that the G55E exchange found in *sop2* might possibly affect the interaction of the exosome core complex (EXO9) with homologues RRP6 or with other proteins that might bind to this part of the exosome surface in plants.

While SOP2/RRP4 and SOP3/HEN2 are known components of the nuclear RNA surveillance machinery, SOP1 is a previously uncharacterized protein. Loss of *sop1* in *pas2-1* background results in accumulation of the *PAS2-1*^*LONG*^ isoform comparable to what is observed in *pas2-1 sop2/rrp4* or *pas2-1 sop3/hen2*, suggesting that the underlying mechanism of *pas2-1* suppression is similar in all three suppressor lines. Moreover, loss of *sop1* results in accumulation of certain misprocessed mRNAs and other transcripts, all of which are also targets of HEN2 and the exosome. Lastly, *sop1* enhances transgene PTGS, as previously observed for *rrp4* and *hen2* [14,27]. Collectively these findings indicate that SOP1 participates in exosome-mediated RNA degradation, which is consistent with its colocalization with HEN2 in nucleoplasmic speckles. However, not all of the targets detected in *hen2* or exosome mutants accumulate also in *sop1* mutants, suggesting that SOP1 participates in the degradation of only a subset of exosome targets. This idea is further supported by the fact that *sop1* has a rather mild effect on PTGS when compared to *sop2/rrp4* or *sop3/hen2*, and that the subcellular localization of SOP1 is restricted to nucleoplasmic speckles while HEN2 and RRP4 are also detected throughout the nucleoplasm and in the entire nucleus, respectively.

The recognition of RNA substrates by the yeast exosome is thought to involve so-called adaptor proteins. For example, the recognition of specific nucleolar RNA targets by the yeast exosome is mediated by the association of the HEN2-related RNA helicase MTR4 with Nop53 for the processing of pre-5.8S rRNA and UTP18 for the degradation of rRNA maturation by-products [42]. Similarly, two ZnF proteins have recently been shown to assist exosome-mediated RNA degradation in *Schizosaccharomyces pombe*. *S*. *pombe* possesses a functional homologue of Arabidopsis HEN2, named Mtl1 (for MTR4-like1), which interacts with the large Zn Finger protein Red1 in the so-called Mtl1-Red1 core of the NURS/MTREC (for Nuclear RNA silencing/Mtl1-Red1-core) complex [43–46]. Another submodule of NURS is the CBCA complex comprising the Cap-binding complex and Ars2 [45,46]. Futhermore, NURS comprises Iss10–Mmi1 and Pab2–Rmn1-Red5, the latter of which is also a Zn-Finger protein [44–46]. Interestingly, NURS is detected in nuclear speckles in *S*. *pombe*, resembling the localisation of HEN2/SOP3 and SOP1 in plants [44,45]. Similar to Arabidopsis HEN2, *S*. *pombe* Mtl1 is required for the exosome-mediated degradation of cryptic unstable transcripts, non-coding RNAs and misprocessed mRNAs [14,46,47] i.e. virtually all types of nuclear exosome substrates. In addition, *S*. *pombe* NURS mediates the elimination of meitotic mRNAs during mitosis [43–45]. The molecular basis for the recognition of meiotic trancripts in *S*. *pombe*, called Determinant for Selective Removal (DSR), has been identified as a repeated consensus sequence U(U/C)AAAC present in introns or 3’UTR [48,49]. Recently, Mmi1 has been shown to be co-transcriptionally recruited to unspliced transcripts containing the UNAAAC consensus sequence in retained introns [50]. No obvious DSR-like sequence was identified in SOP1-targets, such as PAS2-1^LONG^ or AtU2AF65a shown to accumulate in *sop1*. The accumulation of SOP1 targets was shown by qRT-PCR in oligo-dT primed cDNA, indicating that targets SOP1 are oligoadenylated, as is the case for other targets of the nuclear exosome and HEN2 [14,51]. However, it is still unclear whether polyadenylation is a prerequisite of target recognition, or rather a consequence of target accumulation in absence of efficient degradation. Hence, the RNA features that are recognized by SOP1 remain to be identified.

Human and plant nuclear exosome targeting complexes show both common and distinct features when compared to the NURS complex in *S*. *pombe*. While humans have only a single homologue of the RNA helicase MTR4, both *S*. *pombe* and Arabidopsis employ two related RNA helicases in nucleolar and nucleoplasmic degradation processes. In contrast, the NEXT complexes that have been co-purified from humans and plants appear to be rather similar, as they contain related Zn-knuckle and RNA binding proteins [10,14], while sequence homologues of *S*. *pombe* Red1 or Red5 have not been found in plant or human exosome purifications as yet. In *S*. *pombe*, recruitment of Red1 to the exosome core complex requires RRP6 [46]. Although Arabidopsis has three RRP6-like proteins, to date none of them has been shown to interact with the exosome complex and we were not able to identify a sequence homologue of Red1 in Arabidopsis. By contrast, sequence comparison has identified SOP1 as the closest Arabidopsis homologue of *S*. *pombe* Red5, although the sequence homology is restricted to the Zn-Finger domain. The other domains present in SOP1 do not show similarity to known proteins outside plants. Whether SOP1 associates with other protein factors involved in the degradation of nuclear exosome targets remains to be studied.

The link between the exosome, targeting complexes involved in substrate recognition such as NEXT or NURS, and the CAP-binding complex is clearly conserved in *S*. *pombe*, humans and plants [10,14,45,47,52,53]. In humans and *S*. *pombe*, CBC is bound to Ars2, the Arabidopsis homologue of which, named Serrate, was implicated in RNA splicing and the degradation of unspliced mRNA and introns [54,55]. However, in *S*. *pombe*, the physical link between the exosome and the splicing machinery could also be mediated by a direct interaction of the RNA helicase Mtl1 with the spliceosome [46]. Interestingly HEN2, the plant homologue of Mtl1, was co-purified with MagoNashi, a component of the exon-exon junction complex deposited by the splicing machinery, while SOP1 was not yet detected in purifications of plant NEXT-like complexes [14]. It is therefore possible that parallel mechanisms, only some of which require SOP1, enable recognition and degradation of misspliced mRNAs in plants.

## Materials and Methods

### Plant material and growth

*Arabidopsis thaliana* Columbia (Col 0) accession was used throughout this study. Seedlings were grown on Arabidopsis medium [56] supplemented with 1% sucrose in long day condition (16h light) at 18–20°C. The suppressor screen was been performed on EMS-mutagenized individual *pas2-1*^Y^ seeds. The progeny of 800 individual M1 plants were screened on petri dishes for restoration of cotyledons organogenesis on 7-day-old seedlings. The *upf1-5*, *upf3-1* and *ton2-12* mutants have been described previously [23,27,57]. *sop1-5* (salk_019457) and *pas2-4* (GABI_700G11) were obtained from the Nottingham Arabidopsis Stock Center.

### Acyl-CoA analysis

Acyl-CoAs were extracted as described by [58] from 12-say old seedlings frozen in liquid nitrogen, and analysed using LC-MS/MS + MRM in positive ion mode. The LC-MS/MS + MRM analysis (using an ABSciex 4000 QTRAP Framingham, MA) was performed as described by [59], (Agilent 1200 LC system; Gemini C18 column (Phenomenex, Torrance, CA), 2 mm inner diameter, 150 mm length, particle size 5 μm). For the identification and calibration, standard acyl-CoA esters with acyl chain lengths from C14 to C20 were purchased from Sigma as free acids or lithium salts.

### Constructs and plant transformation

*SOP1* genomic DNA was amplified from JAtY54C19 using Phusion polymerase (Life Technologies) and cloned in pDNR207 using Gateway Technology (Invitrogen). *SOP1-GFP* or *SOP1-RFP* fusions were generated by LR recombination in pMDC83 [60] or pH7RGW2 [61]. *RRP4* cDNA in pDNR201 and *RRP4-GFP* have been described previously [27]. *RRP4-RFP* has been generated by LR reaction into pH7RWG2. *HEN2-GFP* has been described previously [14]. *PAS2-1* isoforms were cloned by RT-PCR from *pas2-1* mRNA into pDNR207 by Gateway BP reaction (Invitrogen). PAS2^WT^ cDNA was published in [16]. The various *PAS2* isoforms were cloned in a modified pB7FWG2 vector [61] carrying a 2Kb PAS2 promoter cloned in place of the 35S promoter (SpeI / HindIII). Plant transformations were performed using Agrobacterium C58 pMP90 by the floral dip method [62]. All primers used for construct cloning and plant genotyping are listed in S2 Table.

### Genomic DNA sequencing and SNP determination

Total genomic DNA isolated from whole 12 day old seedlings was extracted using the DNeasy Plant mini kit (Qiagen) according to the manufacturer’s instructions. For genome sequencing of *sop1-1*, DNA was prepared into indexed fragment libraries with amplification and sequenced on an Illumina GAIIx instrument to a minimum of 30 M reads per sample, each with 76 nt read length. Using a custom Perl script, reads were trimmed to 65 nts to remove ends of biased composition and low quality. Reads were mapped to the TAIR10 genomic reference (www.arabidopsis.org) using GenomeMapper in the SHORE software suite [63]. Single nucleotide polymorphism (SNP) variants were determined using SHORE version 0.6 using a consensus minimum coverage of 3 reads. Overlap of SNPs with known genomic features, and functional consequences of SNPs were computed and summarized using FEATnotator [64]. Sequencing of *sop2-1* and *sop3-1* were performed using Illumina Technology (The Genome Analysis Center, Norwich), and mutations were identified using the MutDetect pipeline [65].

### RNA sequencing and analysis

Total RNA were extracted from 12-day-old seedlings using RNeasy extraction kit (Qiagen) according to the manufacturer’s instructions. Reverse transcriptions were performed on 1μg RNA using reverse transcriptase (Fermentas). Quantitative Real-Time PCR (RT-qPCR) reactions were performed as in [14] and Northern Blot as in [15]. For transcriptome analysis, mRNA was enriched from total RNA using oligo(dT) capture (Invitrogen) and prepared into Illumina RNASeq libraries according to the manufacturer’s instructions. Sequencing was performed as paired reads of length 2 x 100 nt on an Illumina GAIIx instrument to minimum depth of 25 M read pairs (50 M reads) per sample. These were trimmed to 88 nt as above and mapped to the Arabidopsis TAIR10 genome reference using Tophat v 2.0.5 [66], with only uniquely mapped reads retained for further analysis. Read number aligned to annotated exon regions (TAIR10) for each annotated gene was computed using a custom Perl script. For genes with multiple isoforms, exons from the representative gene model (TAIR10) were used.

Differential expression between samples was analyzed pairwise using NOISeq ver. 2.0.0 [67], an R bioconductor package that uses read count data as input. NOISeq was used to simulate 5 samples within each condition (nss parameter), permitting 0.2% of total reads in each condition for each simulated sample (pnr parameter) and a variability (v parameter) of 0.02 in total sequencing depth of simulated samples. Normalization (norm parameter) was according to the RPKM calculation, and for genes with zero read counts, a pseudo count of 0.5 was used (k parameter) for computing RPKM. Correction factor for length normalization (lc parameter) was set to 1, indicating counts to be divided by a single order of length. The NOISeq pipeline was repeated for exons alone, and for full length genes (both exons and introns included).

RNAseq reads have been deposited in the NCBI short read archive (SRA) under the accession numbers listed in the BioProject PRJNA293799.

### PTGS assay

GUS activity was quantified as described before [68] using crude extracts from plant leaves and monitoring the quantity of 4-methylumbelliferone products generated from the substrate 4-methylumbelliferyl-b-D-glucuronide (Duchefa) on a fluorometer (Thermo Scientific fluoroskan ascent).

### Microscopy

Imaging of fluorescent fusion proteins was performed on 7 day-old roots by confocal scanning laser microscopy on a Zeiss LSM710 microscope equipped with a 63X 1.20 NA water-immersion objective. Excitation of fluorophore were performed at 488nm for GFP and 561nm for RFP and emission settings were 500–550nm for GFP and 570–620nm for RFP. Multichannel confocal stacks were processed with ImageJ 1.49h for figure preparation.

### Data availability

The raw data of *sop1-1* transcriptome analysis by RNAseq have been deposited to NCBI short read archive (SRA) accessible in the BioProject PRJNA293799. Data are also available in a user-friendly Jbrowse interface at http://sop1rna.inra.fr

## Supporting Information

S1 Fig Phenotypes of the isolated *sop* alleles. (A) Growth phenotypes of 7-day-old seedlings (top) or 5-week-old plants (bottom) of the various *sop* mutants isolated in the *pas2-1* suppressor screen. Bar = 5mm. (B) Chromatogram showing the Acyl-CoA profile of the various genotype studied. The blue star indicates 3-hydroxylated acyl-CoA synthesis intermediates accumulating in *pas2-1* mutants.
(TIF)

S2 Fig *sop1-5* mutation disrupts *SOP1* mRNA but does not affect *PAS2* transcript accumulation. (A) Gene structure of *SOP1* and *PAS2* genes showing the position of *sop1-5* TDNA insertion and the amplicon used for the RT-qPCR presented in B. (B) RT-qPCR of *SOP1* and *PAS2* in *sop1-5* compared to wild type (Col 0).
(TIF)

S3 Fig *pas2-1* mutation results in the production of mRNA isoforms. (A) *PAS2* (At5g10480) gene structure with exons displayed as colored boxes. The red star indicates *pas2-1* mutation, blue arrows represent the primer used for RT-PCR in B. (B) Electrophoresis of *pas2-1* cDNA after RT-PCR displaying 3 bands containing the 4 PAS2 RNA isoforms. (C) Sequences corresponding to the cloned ends of the various PAS2 isoforms. Color coding of the sequences correspond to the different exons shown in A. The *pas2-1* SNP is shown in red and the last intron in black. Consensus sequences used to identify isoform-specific reads in the RNAseq are highlighted in bold letters.
(TIF)

S4 Fig Intron retention does not lead to systematic transcript accumulation in *sop1-1* mutant. Normalized absolute values of reads mapped to the At5g36880 (ACS) and At5g10480 (PAS2) loci in wild type (Col), *pas2-1*^*Y*^ and *pas2-1*^*Y*^*sop1-1*. The gene organisation with introns and exons is shown at the top of each panel with grey/blue lines for introns and red boxes for exons, red lines representing UTRs. The directionality of transcription is indicated by the black arrow at the end of the gene. Sequences of the exon-intron junction affected are displayed below with the mutated nucleotide in red. The intron-retention events are highlighted by a red arrow. Note that, unlike for *PAS2*, the intron retention in *ACS* is not associated with transcript accumulation in *pas2-1*^*Y*^*sop1-1* compared to WT or *pas2-1*^*Y*^.
(TIF)

S5 Fig Complementation of *pas2-1 sop* mutants by GFP fusion proteins. (A) Complementation of *pas2-1*^*Y*^*sop1-1* phenotype by expression of 35S::SOP1-GFP or 35S::SOP1-RFP. (B) Complementation of *pas2-1*^*Y*^*sop2-1* phenotype by expression of 35S::RRP4-GFP or 35S::RRP4-RFP. (C) Partial complementation of pas2-1^Y^sop3-1 phenotype by expression of 35S::HEN2-GFP. (D) Confocal laser scanning imaging of root cells from plants stably expressing SOP1, SOP2 and SOP3 proteins in fusion with GFP. (bar = 5μm) (E) Confocal laser scanning imaging of root cells from plants stably co-expressing SOP1-RFP (red channel) and HEN2-GFP (green channel). The white transparent arrow represents the line analyzed as a kymograph in panel (F). (F) Kymograph representation of the foci’s movement along the curved arrow highlighted in (E). Foci dynamic is presented in S2 Movie.
(TIF)

S6 Fig *sop* mutants, but not the NMD mutants, share accumulation of mispliced SOP1 targets. (A) Phenotype of 7-day-old seedlings of the indicated genotype. (B) RT-PCR of SOP1-target genes U2AF65a and SRP30 in the genotypes shown in A.
(TIF)

S1 Movie SOP1-GFP localization in root epidermal cells. Left panel is a frame-by-frame representation of an image stack through a root epidermal cell of a wild type plant expressing SOP1-GFP (green) stained with propidium iodide (red). Right panel corresponds to a 3D rendering of the SOP1-GFP signal of the same cell.
(AVI)

S2 Movie Dynamic of SOP1 and HEN2 nucleoplasmic foci in root epidermal cells. Imaging every 15 seconds over a period of 10 minutes of root epidermal cells expressing SOP1-RFP (red) and HEN2-GFP (green). An overlay of the two channels is presented on the right panel with SOP1 and HEN2 colocalization displayed in yellow.
(AVI)

S1 Table List of genes identified in the RNAseq analysis presented in Fig 6A and 6B. (XLSX)

S2 Table Primers used in this study. (XLSX)
