# Correction: The Zinc-Finger Protein SOP1 Is Required for a Subset of the Nuclear Exosome Functions in Arabidopsis

**DOI:** 10.1371/journal.pgen.1005958

**Published:** 2016-03-23

**Authors:** Kian Hématy, Yannick Bellec, Ram Podicheti, Nathalie Bouteiller, Pauline Anne, Céline Morineau, Richard P. Haslam, Frederic Beaudoin, Johnathan A. Napier, Keithanne Mockaitis, Dominique Gagliardi, Hervé Vaucheret, Heike Lange, Jean-Denis Faure

S2 Table is incomplete. Please view the complete S2 Table here.

There is an error in the third subsection of the Results section, entitled “*sop1* specifically suppresses the splicing-defective *pas2-1* allele”. The sentence “For this purpose, we introgressed *sop1-5*, a knock-out allele, that harbours a T-DNA insertion in the At5g21580 locus which encodes the SOP1 protein (see below), into the original *pas2-1* mutant as well as into *pas1-2*, *pas2-4* and *pas3-1* mutants [16,21,22].” should read: “For this purpose, we introgressed *sop1-5*, a knock-out allele, that harbours a T-DNA insertion in the At1g21580 locus which encodes the SOP1 protein (see below), into the original *pas2-1* mutant as well as into *pas1-2*, *pas2-4* and *pas3-1* mutants [16,21,22].”

## Supporting Information

S2 Table Primers used in this study. (XLSX)
