# Supplementary figures and images for: The Zinc-Finger Protein SOP1 Is Required for a Subset of the Nuclear Exosome Functions in Arabidopsis
Source: PLoS Genet. 2016 Feb 1;12(2):e1005817. doi: 10.1371/journal.pgen.1005817 (PMC4735120; doi:10.1371/journal.pgen.1005817)

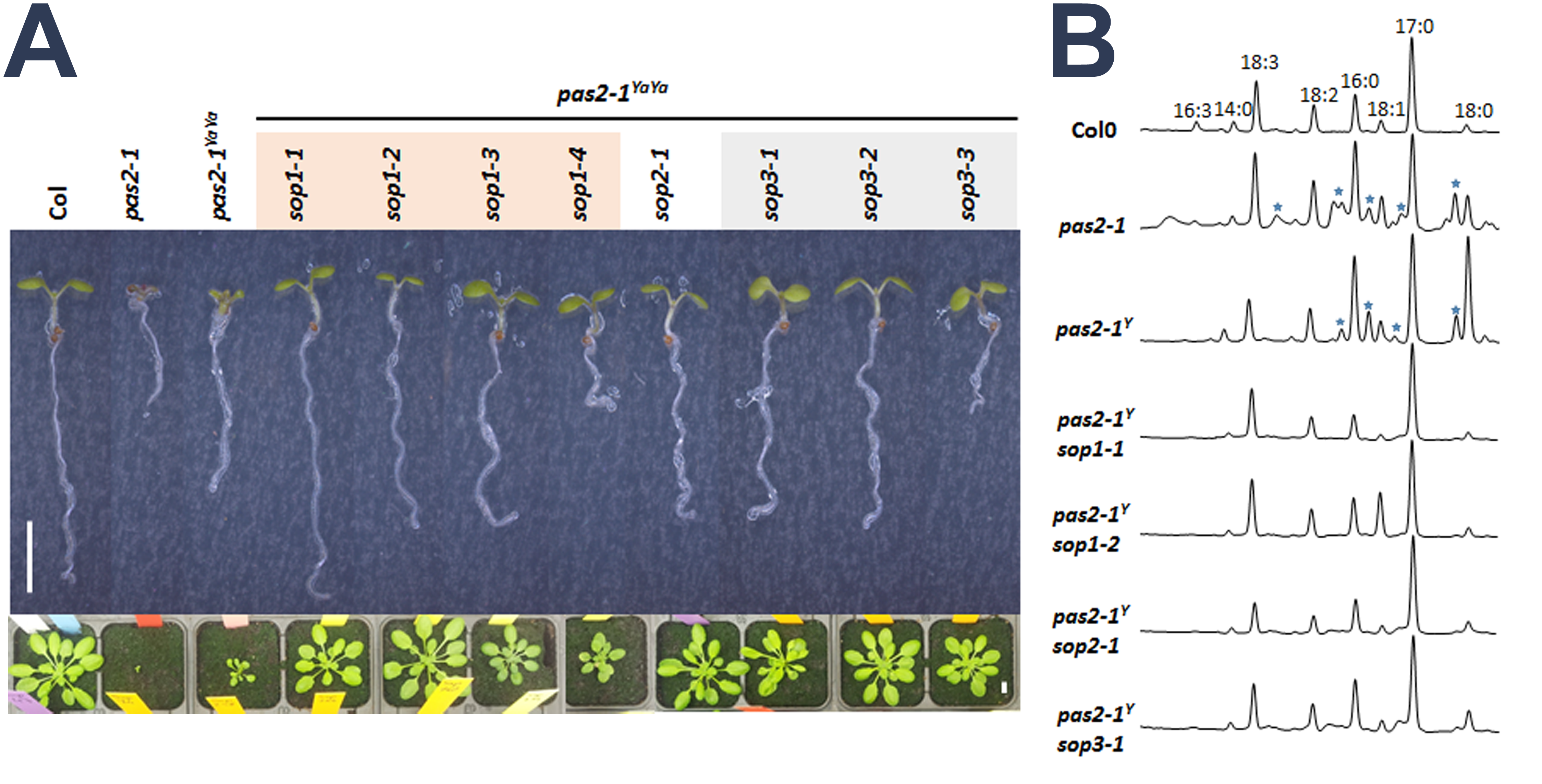

Supplement: S1 Fig — (A) Growth phenotypes of 7-day-old seedlings (top) or 5-week-old plants (bottom) of the various sop mutants isolated in the pas2-1 suppressor screen. Bar = 5mm. (B) Chromatogram showing the Acyl-CoA profile of the various genotype studied. The blue star indicates 3-hydroxylated acyl-CoA synthesis intermediates accumulating in pas2-1 mutants. (TIF) [file pgen.1005817.s001.tif]

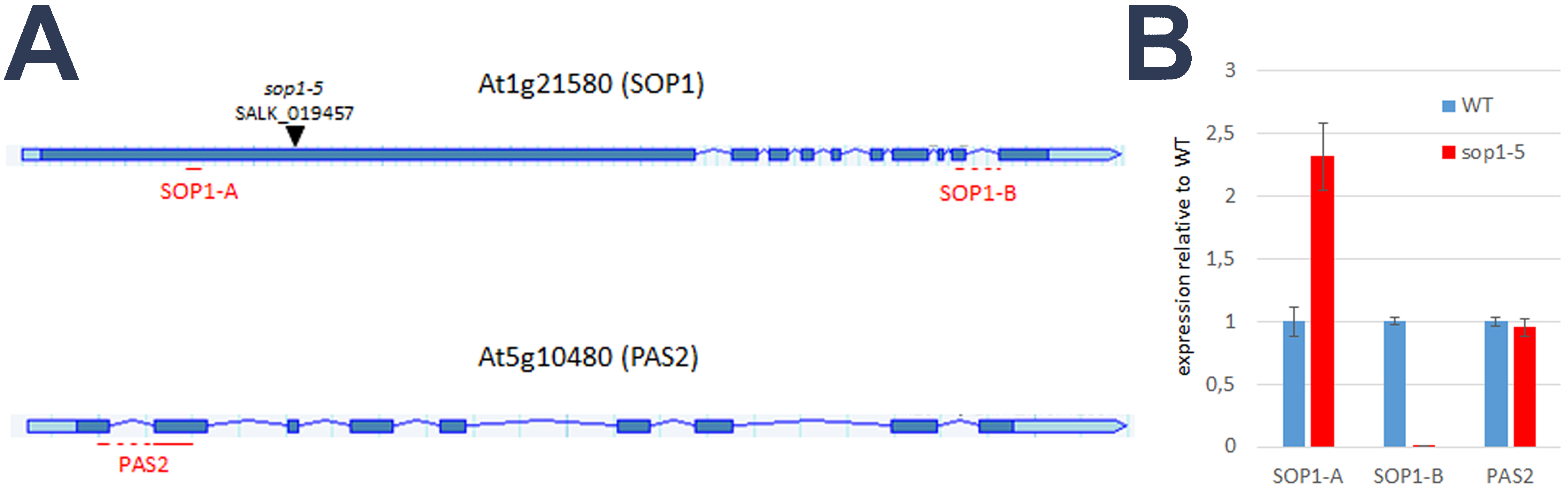

Supplement: S2 Fig — (A) Gene structure of SOP1 and PAS2 genes showing the position of sop1-5 TDNA insertion and the amplicon used for the RT-qPCR presented in B. (B) RT-qPCR of SOP1 and PAS2 in sop1-5 compared to wild type (Col 0). (TIF) [file pgen.1005817.s002.tif]

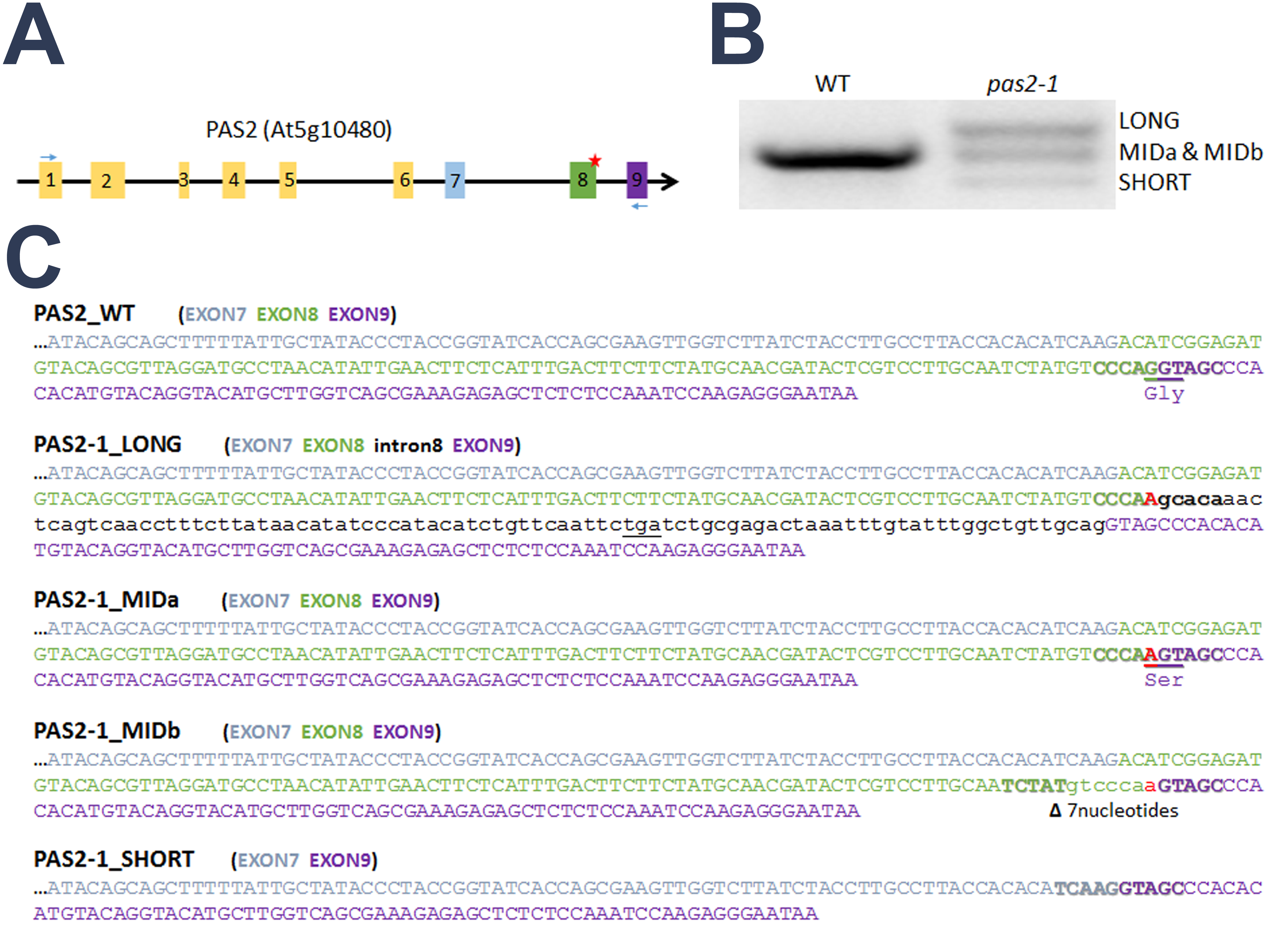

Supplement: S3 Fig — (A) PAS2 (At5g10480) gene structure with exons displayed as colored boxes. The red star indicates pas2-1 mutation, blue arrows represent the primer used for RT-PCR in B. (B) Electrophoresis of pas2-1 cDNA after RT-PCR displaying 3 bands containing the 4 PAS2 RNA isoforms. (C) Sequences corresponding to the cloned ends of the various PAS2 isoforms. Color coding of the sequences correspond to the different exons shown in A. The pas2-1 SNP is shown in red and the last intron in black. Consensus sequences used to identify isoform-specific reads in the RNAseq are highlighted in bold letters. (TIF) [file pgen.1005817.s003.tif]

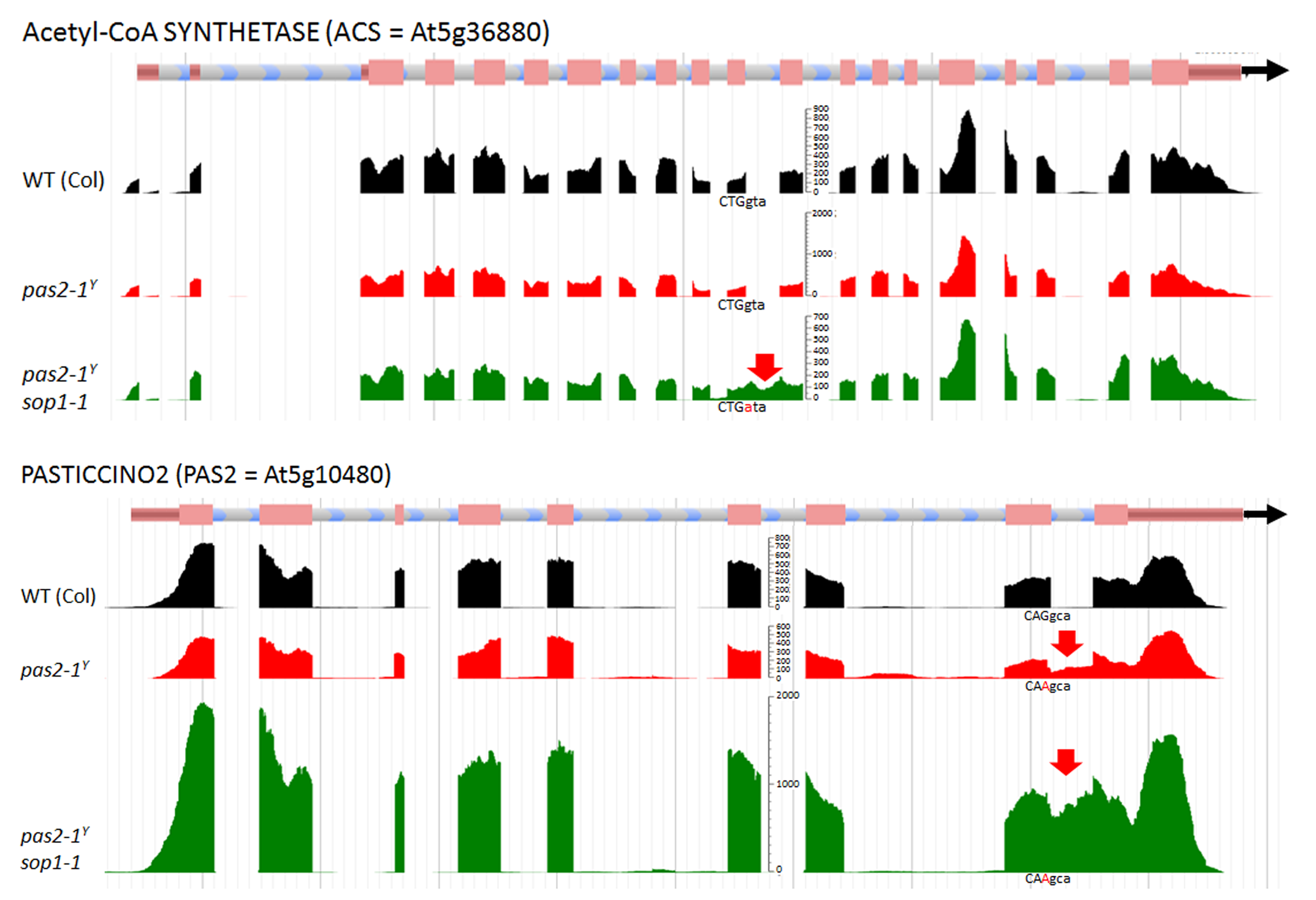

Supplement: S4 Fig — Normalized absolute values of reads mapped to the At5g36880 (ACS) and At5g10480 (PAS2) loci in wild type (Col), pas2-1Y and pas2-1Ysop1-1. The gene organisation with introns and exons is shown at the top of each panel with grey/blue lines for introns and red boxes for exons, red lines representing UTRs. The directionality of transcription is indicated by the black arrow at the end of the gene. Sequences of the exon-intron junction affected are displayed below with the mutated nucleotide in red. The intron-retention events are highlighted by a red arrow. Note that, unlike for PAS2, the intron retention in ACS is not associated with transcript accumulation in pas2-1Ysop1-1 compared to WT or pas2-1Y. (TIF) [file pgen.1005817.s004.tif]

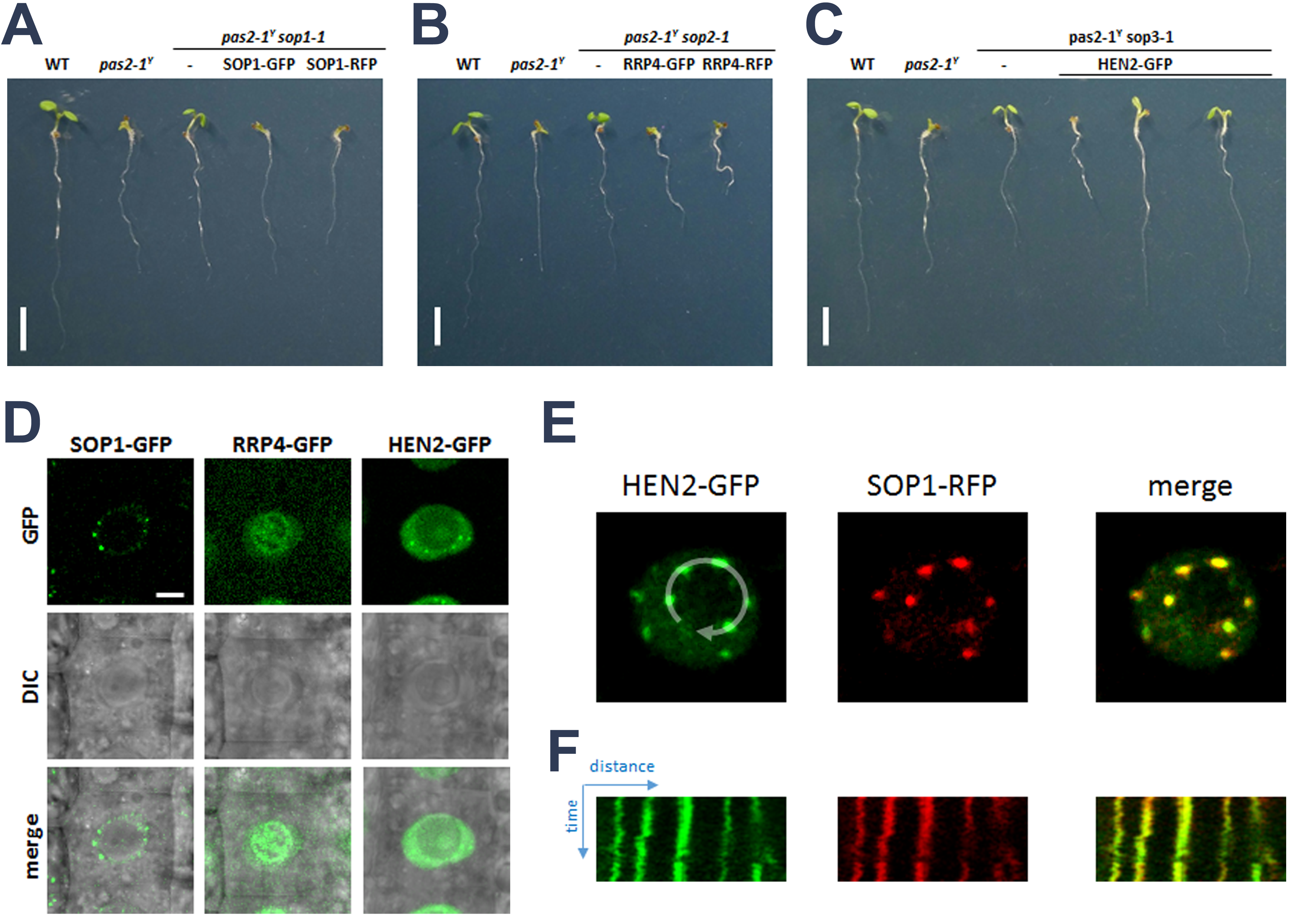

Supplement: S5 Fig — (A) Complementation of pas2-1Ysop1-1 phenotype by expression of 35S::SOP1-GFP or 35S::SOP1-RFP. (B) Complementation of pas2-1Ysop2-1 phenotype by expression of 35S::RRP4-GFP or 35S::RRP4-RFP. (C) Partial complementation of pas2-1Ysop3-1 phenotype by expression of 35S::HEN2-GFP. (D) Confocal laser scanning imaging of root cells from plants stably expressing SOP1, SOP2 and SOP3 proteins in fusion with GFP. (bar = 5μm) (E) Confocal laser scanning imaging of root cells from plants stably co-expressing SOP1-RFP (red channel) and HEN2-GFP (green channel). The white transparent arrow represents the line analyzed as a kymograph in panel (F). (F) Kymograph representation of the foci’s movement along the curved arrow highlighted in (E). Foci dynamic is presented in S2 Movie. (TIF) [file pgen.1005817.s005.tif]
